# Transparent Soil for Imaging the Rhizosphere

**DOI:** 10.1371/journal.pone.0044276

**Published:** 2012-09-11

**Authors:** Helen Downie, Nicola Holden, Wilfred Otten, Andrew J. Spiers, Tracy A. Valentine, Lionel X. Dupuy

**Affiliations:** 1 The James Hutton Institute, Invergowrie, Dundee, United Kingdom; 2 The SIMBIOS Centre, University of Abertay Dundee, Bell Street, Dundee, United Kingdom; University of Nottingham, United Kingdom

## Abstract

Understanding of soil processes is essential for addressing the global issues of food security, disease transmission and climate change. However, techniques for observing soil biology are lacking. We present a heterogeneous, porous, transparent substrate for *in situ* 3D imaging of living plants and root-associated microorganisms using particles of the transparent polymer, Nafion, and a solution with matching optical properties. Minerals and fluorescent dyes were adsorbed onto the Nafion particles for nutrient supply and imaging of pore size and geometry. Plant growth in transparent soil was similar to that in soil. We imaged colonization of lettuce roots by the human bacterial pathogen *Escherichia coli* O157:H7 showing micro-colony development. Micro-colonies may contribute to bacterial survival in soil. Transparent soil has applications in root biology, crop genetics and soil microbiology.

## Introduction

The ability of plants and microorganisms to successfully exploit soil resources underpins the survival of all terrestrial life. Soil is a complex assemblage of mineral and organic particles that can host a very diverse range of biological organisms [1]. It comprises a solid phase, consisting of minerals and organic matter particles, and an aqueous phase with dissolved minerals and gasses essential for plant nutrition and microbial activity. In non-saturated soil, air is also available in large pores supplying gasses required for metabolic processes of plants and microbes. Imaging technologies are required to study and quantify soil biological processes [2], but this is difficult because of the inherent opacity of soil. Non-optical imaging techniques have been used to image plant roots in soil, for example, x-ray microtomography and MRI [3], [4], but these methods are not adapted for imaging biological activity, mostly because of the inability to use them to detect common fluorescent markers.

Biological studies are benefiting immensely from emerging optical imaging technologies. For example, Optical Projection Tomography (OPT), which uses a collection of 2D projections to reconstruct 3D volumes, has allowed the distribution of fluorescent markers to be mapped across intact whole embryos [5]. Recent advances in Selective Plane Illumination microscopy (SPIM) have allowed the light doses received by samples to be drastically reduced during live imaging. Using this technique, it was possible for the first time to track individual cell growth and cell division events across entire embryos during 24 hours [6]. It is now also possible to overcome diffraction limitations and increase resolution with techniques such as 3D structured illumination microscopy. This is now a common technique to observe sub-cellular processes [7].

Unfortunately, the study of soil biota is not benefiting much from technological advances in optical imaging because most soil organisms, such as many types of fungi, cannot be cultured in current artificial substrates, whilst others have their functions strongly affected by the medium they are grown in [8]. We have developed a substrate called transparent soil, with a matrix of solid particles and a pore network containing liquid and air. The physical structure was manipulated with the aim of generating 3D optical images of soil biota in a physically complex yet controllable environment.

## Results and Discussion

### Making Soils Transparent using Refractive Index Matching

**Figure 1:**
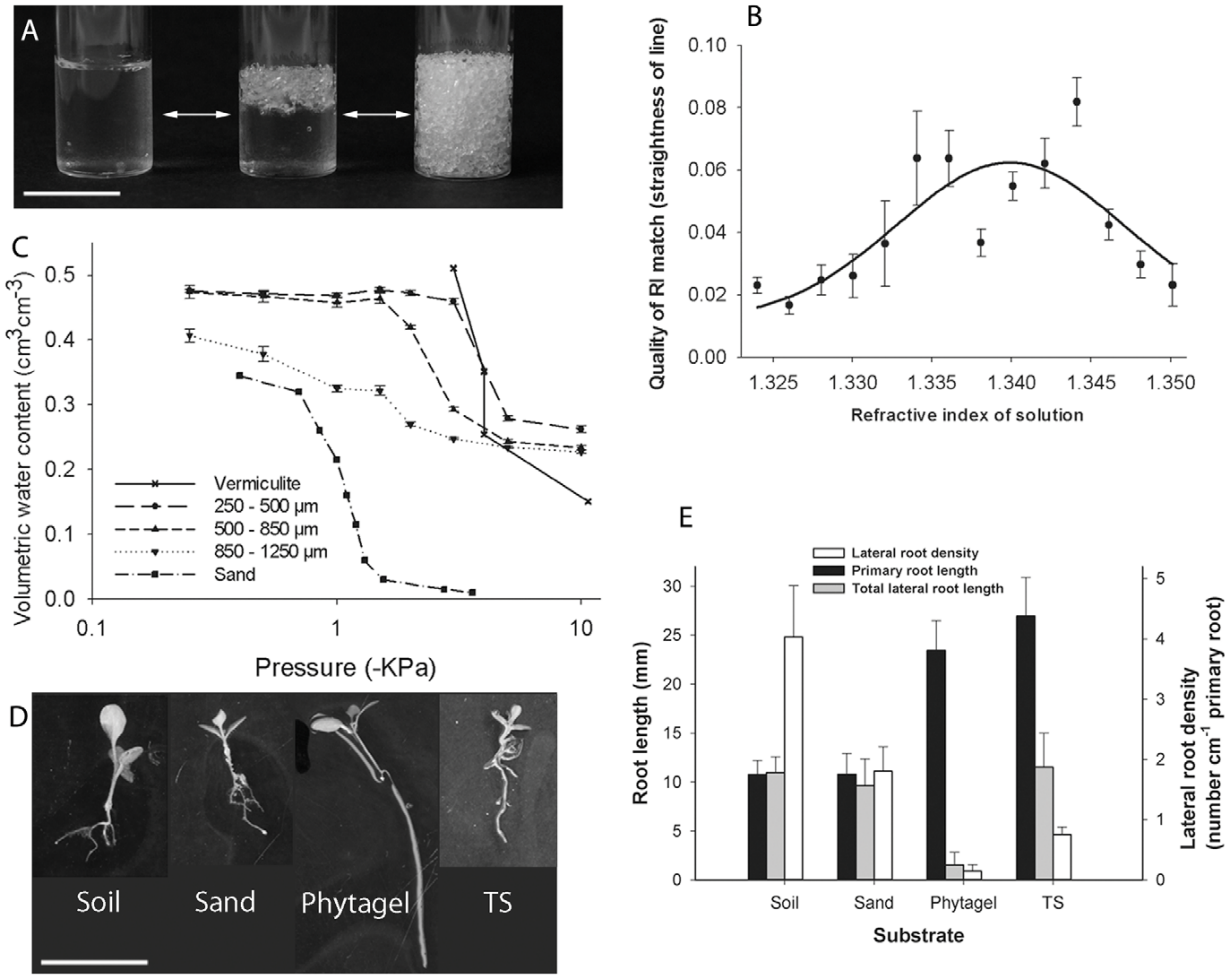
Characterisation of the transparent soil. A. Transparent soil is prepared for imaging by saturation with RI-matched solution to achieve transparency (left, fully saturated; right, larger pores are drained). Scale bar = 2.5 cm. B. Optimal RI of nutrient solution for RI matching with Nafion using projected straight line images deformed by the substrate. Curve shows gaussian non-linear regression (R^2^ = 0.38). C. Water retention in transparent soil with 3 different Nafion particle sizes compared to vermiculite [37] and sand [16]. Error bars show standard error. D–E. Comparison of plant growth in transparent soil and other substrates. D. Excavated plants with representative root systems from each substrate type after 2 weeks of growth. Scale bar represents 1 cm. E. Quantification of root system parameters in different substrates. Plants grown in transparent soil had lateral root lengths and densities more similar to plants grown in soil than plants grown in phytagel.

At the boundary of two transparent materials with different refractive indices, the path of light is distorted through refraction. By matching the refractive index (RI) of a solid and a liquid, this effect is negated so that the boundaries between the materials become invisible. RI matching has proved a powerful approach in many areas of physical sciences, such as fluid dynamics [9] and colloid sciences [10]. In soil mechanics, amorphous silica particles have been used with oil-based RI matching solutions [11] and have similar mechanical properties to clay [12]. This system has been used for investigating particle displacement in response to the application of mechanical forces. Recently, the technique of RI matching has been adapted for growing and imaging aquatic biofilms [13] where limited RI matching was achieved using water. In the present study, we have used particles of Nafion, which has a low refractive index (1.34), close to that of water (1.33), and therefore allows RI matching with an aqueous solution (Fig. 1A,B). During the period of plant and microbial growth, pores were partially saturated with a plant nutrient solution [14] and air spaces were maintained for aerobic respiration. Roots can grow freely in 3D trajectories whilst responding to heterogeneous, complex touch stimuli, replicating the mechanical processes that would occur in soil [15]. Immediately before imaging, the substrate was saturated using a RI matched liquid plant nutrient solution so that imaging of roots and microorganisms could be carried out in situ (Fig. 1A).

### Mimicking Physical and Chemical Properties of Soil

We have also sought to mimic physical and chemical properties important for supporting plant and microbial growth in soils in the transparent soil system. Nafion, the building block of transparent soil, is a transparent ionomer (synthetic polymer with ionic properties) that is physically and chemically adaptable. Nafion particle size distribution has been manipulated by freezer milling (250–1600 µm). We have also altered the surface chemistry of the particles in order to control water retention and nutrient availability through changes in ion exchange capacity. We analysed the water retention of transparent soil with 3 particle size categories and compared this to vermiculite and sand (Fig. 1C). In the 2 smallest size categories, and in vermiculite, the sharpest release of water occurred between −1.5 and −5 kPa. The water release in the largest sized particles was more gradual but in all sizes, a levelling off of water release occurred towards −10 kPa and the residual water content measured in transparent soil ranged from 0.23 to 0.26 cm^3^ cm^−3^. This value was higher than is usual in sand [16], despite the similarity in particle size. Nafion has a complex structure incorporating networks of hydrophilic channels that allow transport of water and other polar solvents [17]. Although the exact nature of these networks is still unclear, it is estimated that the diameter of these channels varies between 1 and 6 nm [18], [19], [20]. At this range of scales, the hydrogen bonds holding the water molecules are extremely strong and the water sorbed in the Nafion particles cannot be accessed by most biological organisms.

Cation exchange is an important characteristic of natural soils, particularly in those containing clay, which acts as a buffer for minerals in the soil solution. The cation exchange capacity (CEC) of transparent soil was quantified as 81 meq100 g^−1^. This is within the range that could be expected for vermiculite (80–150 meq100 g^−1^ [21]). Additionally, anion exchange in soils involves some essential plant nutrients such as nitrate and phosphate, which are of strong interest in plant nutrition. The anion exchange capacity (AEC) of cationic Nafion [22] was 47 meq100 g^−1^.

### Root Growth in Transparent Soil

Transparent soil can be used for a large range of other applications. At the macroscopic level, quantifying the growth of root systems is essential in understanding how plants obtain resources. To analyse root growth in transparent soil, we have measured primary root length, total root length and root diameter of root systems of plants grown on phytagel, sandy loam soil, sand, and transparent soil. The Analysis of Variance showed the type of substrate had a significant effect on root length (p≤0.003) (Fig. 1E) and root diameter (p = 0.026). The mean root diameter was 0.24±0.01 mm in soil, 0.24±0.03 mm in sand, 0.18±0.02 mm in phytagel and 0.28±0.03 mm in transparent soil. The least significant difference test showed that plants grown on transparent soil, soil, or sand had more lateral roots and a higher biomass than plants grown on phytagel (a common substrate used for growing plants and imaging roots). Root systems from soil and sand had shorter primary roots than plants grown on gel and transparent soil, but plants grown in phytagel had long primary roots and almost no laterals (Fig. 1D,E). Our results indicate that root growth in transparent soil is similar to that observed in soil and sand and demonstrate the importance of physical heterogeneity in the growth substrate for producing soil-like root systems.

**Figure 2:**
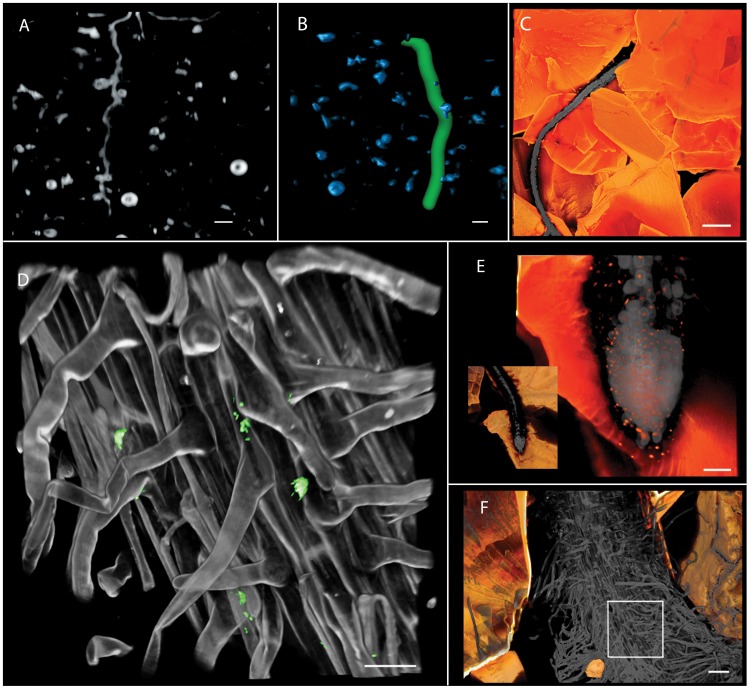
Imaging roots and microorganisms in transparent soil using OPT and confocal microscopy. A. Projection image from OPT scan of *Nicotiana benthamiana* roots. Scale bar represents 1 mm. B. Root tracking algorithm is applied to the reconstructed data to segment and dilate (to improve visibility) the root (green) from the small air bubbles (blue). Scale bar represents 1 mm. C–F. Snapshots of volume renderings of confocal scans. C. *Arabidopsis thaliana* roots expressing GFP in plasma membranes (grey) in transparent soil with sulphorhdamine-B-dyed particles (orange) where scale bars represents 300 µm. Inset shows root skeletonisation and edge detection applied to scan C to detect roots and particles. D. GFP labelled *Escherichia coli* 0157:H7 cells and colonies on surface of *Latuca sativa* (lettuce) root with prominent root hairs. Scale bar represents 30 µm. E. Box shows enlarged region of lettuce root in D with Nafion particles visible in orange. Scale bar represents 100 µm. F. *Arabidopsis thaliana* root tip with nuclear RFP expression linked to auxin reporter [41]. Inset with box shows enlarged region. Scale bar represents 54 µm.

Transparent soil is suitable for growing and imaging the roots of various plant species, including alfalfa, barley, maize (data not shown), tobacco, lettuce and thale cress (*Arabidopsis*), and imaging at the whole root level can be achieved using OPT (Fig. 2A). Transparent soil provides images with low levels of noise and opens avenues for automated analyses of genetic screens [23]. In addition, the availability of fluorescent signals eases the discrimination of biological structures where separation of the different wavelengths provides much of the segmentation of the biological structures (Fig. 2B,C). Transparent soil can also be used to capture cellular events using plants with plasma membrane and nucleus-localized reporter gene-encoded proteins (Fig. 2C, Fig. S1, Video S1, VideoS2, Video S3), which could be used for automated analysis of multicellular development [24], [25]. For example, we have imaged the 3D distribution of auxin in *Arabidopsis thaliana* root tips (Fig. 2F) using auxin reporter lines [26].

### Application of Transparent Soil to the Study of Root Bacteria Interactions

We have applied transparent soil to study the mechanisms of transmission of food-borne human pathogens on fresh produce plants using GFP-labelled *Escherichia coli* O157:H7. Although this strain is an enteric animal pathogen, it is able to use plants as alternative hosts. The contamination route can be from crop irrigation or manure, via the rhizosphere before entering the human food chain [27]. In order to study the mechanisms of survival of *E. coli* O157:H7 in a soil-like environment, lettuce seeds were germinated and inoculated with *E. coli* O157:H7 before transferring them to the transparent soil. Our results showed that after 7 days of growth, *E. coli* O157:H7 had survived in a soil like environment in the form of micro-colonies of various sizes. Micro-colonies developed in the rhizosphere [28]. Since *E. coli* O157:H7 is not solely a rhizosphere bacteria, the formation of micro-colonies shows an adaptation to the plant host, which will increase survival in the root zone (Fig. 2D, Video S4). As a pathogenic bacteria, *E. coli* O157:H7 survives by colonizing host organisms and in the initial stages of colonization, adheres to the host [29]. When using transparent soil, there is a potential problem of moving the bacteria during saturation of the substrate in preparation for imaging but this should not affect attached bacteria such as *E. coli* O157:H7 in the preliminary stages of colonization. Saturation is, however, a potential limitation of the method if studying microbes that are not attached to surfaces because it is likely that these would be moved during saturation. In summary, our results show that transparent soil is ideal for imaging studies of certain plant-microbe interactions in situ at the microscopic level.

### New Opportunities for Plant Sciences

Soil microbes provide numerous important services [30] and their interactions with plants enhance the supply of nutrients, for example by nodulation [31] or by biological fertilization [32]. The transfer of human pathogens in the food chain [27] and spread of crop diseases [33] also involve complex plant-microbe interactions. The use of transparent soil will facilitate quantitative imaging of the dynamics of in situ root-microbe interactions using high resolution imaging with fluorescence for detecting microorganisms expressing fluorescent proteins (Fig. 2D). For plant genetics and crop breeding, transparent soil could be integrated with high-throughput screening systems for root traits [34] that may improve nutrient acquisition and reduce the need for fertilizers [35]. Overall, this approach presents new opportunities to unravel the complex processes of plant-soil interactions in situ and in vivo and holds promise for a wide range of applications to aid the understanding of important underlying relationships that underpin the sustainability of our ecosystems.

## Materials and Methods

### Construction of Transparent Soil

Nafion was from Ion Power Inc., USA, in the form of 4 mm×3 mm pellets. Acid (NR50 1100) and precursor (R1 100) forms were used. Size reduction of Nafion particles was performed using a freezer mill (6850, SPEX SamplePrep, UK). The final particle size range was 350–1600 µm. Cation exchanging Nafion particles were made by ensuring full conversion to the acid form by washing in a solution of 15% v/v KOH, 35% v/v DMSO and 50% dH_2_O at 80°C for 5 hours, then with dH_2_O (milliQ) at room temperature for 30 minutes followed by several dH_2_O rinses. This was followed by 2 washes in 15% v/v nitric acid at room temperature for 1 hour and then overnight. The particles were treated with 1 M sulphuric acid for 1 hour at 65°C, and the acid was removed and replaced with dH_2_O at 65°C for 1 hour. After cooling, the particles were washed several times with dH_2_O. They were washed in 3 wt % H_2_O_2_ solution at 65°C for 1 hour and allowed to cool. The particles were rinsed again multiple times with fresh dH_2_O [36]. To titrate the particles with mineral ions, stock solutions of MSR media were used to immerse the particles. These were shaken at 30°C for 30 minutes before replacing the nutrient solution. This was repeated until the pH was neutral and stable. The particles were rinsed with dH_2_O to remove excess MSR. Before use, the particles were autoclaved in dH_2_O for sterilisation.

### Refractive Index Matching

To determine the best refractive index match between the particles and liquid, plastic cuvettes were filled with acid Nafion particles and saturated with a range of concentrations of sorbitol solutions from 0–13% (w/v) to achieve a range of refractive indices. On one side of each cuvette, a straight line was drawn from top to bottom and a projection image was taken through solid/liquid mix. There were 5 replicate images taken at each sorbitol concentration at 20°C. The straightness of the line for each image was used as an indicator of the light path distortion by refraction. The thresholded image was skeletonized and a bounding box around the line was created. The straightness was calculated as straightness = height of bounding box/area of bounding box. Nutrient-titrated Nafion particles were also tested in this way, but with a larger range of sorbitol concentrations.

### Characterising Properties of Transparent Soil

Water retention was measured in transparent soil with 3 size categories of Nafion particles (200–500 µm, 500–850 µm and 850 −1250 µm, n = 3), with a dry mass of 10.3±0.1 g. Saturated samples were placed on ceramic plates in glass funnels, which were connected to hanging water columns. Different suctions were achieved by moving the water level in the water column to a specific height. At each pressure, the water content of the sample was allowed to equilibrate and the mass was recorded to allow calculation of volumetric water content. Data on water retention in vermiculite and sand from other studies were used for comparison with our data on water retention in transparent soil [16], [37]. Exchangeable cations were extracted using the ammonium acetate method [38] and cation exchange capacity was quantified by subsequent ICP-MS analysis. To measure anion exchange capacity, sorbed chloride ions were exchanged with nitrate ions and exchange capacity was determined by measuring the extracted chloride ions [39]. Chemical analyses were carried out by Macaulay Analytical at The James Hutton Institute.

### Plant Culture

*Arabidopsis thaliana* expressing 35S:LTI6b- EGFP (constitutively expressed *enhanced* green fluorescent protein targeted to the plasma membrane), in the C24 background (originally obtained from Dr. J. Haseloff, University of Cambridge, UK) [40] and auxin reporter lines [41] were used for confocal microscopy. *Nicotiana benthamiana* (tobacco) and *Latuca sativa* (lettuce, var. capitata, Seed Parade, UK) seeds were surface sterilized by washing in 10% bleach for 20 minutes followed by several sterile dH_2_O washes. *Arabidopsis thaliana* seeds were sterilized on filter paper by adding 70% ethanol, allowed to dry slightly and addition of 90% ethanol before allowing to air dry. MSR nutrient media [14] was used for culturing tobacco seeds and half-strength Murashige and Skoog (M&S) basal media (Sigma) was used for lettuce and *Arabidopsis* seeds. Seedlings were germinated before use in experiments by sowing seeds in petri dishes with 5 g L^−1^ phytagel (Sigma) with MSR or M&S nutrient media. Plants were incubated at 20°C with 16 hours light: 8 hours darkness.

### Analysis of Plant Growth in Different Substrates

The substrates used for analysing plant growth were 1. sandy-loam soil from Lower Pilmore field, The James Hutton Institute, Dundee, UK. The soil was sieved to 3 mm and packed to a density of 1.2 g cm^−3^ with a gravimetric moisture content of 20% (n = 9). 2. Horticultural grit sand (Gem, UK), with a dry bulk density of 1.5 g cm^−3^ and MSR to achieve a gravimetric moisture content of 15.2% (n = 9). 3. 4 g L^−1^ phytagel (Sigma) with MSR (n = 9). 4. Transparent soil, prepared as described below and packed to a density of 1.03 g cm^−3^ (n = 6). Growth period was 2 weeks after transferring the seedlings to the media in cylindrical glass sample holders, diameter = 2.5 cm, height 7.5 cm. All plants were excavated, the roots were washed and they were mounted onto acetate sheets for scanning using a flatbed scanner (Epson expression 1640 XL). Primary and lateral roots were measured using the segmented line function from ImageJ software (National Institute of Health, USA).

### Bacterial Culture and Experimental Setup

*Escherichia coli* 0157 : H7 was transformed with a fluorescent reporter plasmid (*loc8*-e*gfp*) [42] and grown in MOPS glucose media with amino acids and chloramphenicol (25 µg µl^−1^) at 18°C with aeration for 20 hours. One day after sowing the lettuce seeds, germination occurred and 15 ml of bacteria suspended in half-strength M&S media at a cell density of 2×10^7^ cfu/ml was used to inoculate the seedlings in a Petri dish, at room temperature, for 30 minutes, before transferring the seedlings into growth chambers with transparent soil, as described above. Imaging was carried out after 5 days after sowing. The method used for bacteria-plant interactions allowed colonization of the roots to develop from infected seedlings, rather than from the addition of the inoculum directly to the substrate or the more mature roots.

### 3D Optical Imaging of Soil Biological Processes

For OPT imaging the samples were prepared in glass cylindrical specimen tubes (2.5 cm in diameter, 7.5 cm in height) with a substrate volume of 15 cm^3^. Duration of growth was dependent on plant species but in general, imaging was performed before the roots reached the base of the tube. Tobacco plants used for OPT were imaged 10 days after sowing. *Arabidopsis* plants used for confocal imaging were imaged 10–14 days after sowing. The OPT setup was built in-house and consists of a light box, stage for sample with rotating stepper motor, stereo microscope (Leica MZ 16 FA) and camera (Leica DFC 350 FX). The stage and camera were controlled by software also built in-house, allowing control of the number of images acquired for each sample. The projection images were reconstructed to produce 3D data using a filtered backprojection algorithm with the Iradon function in Matlab (The MathWorks, Inc.).

For CLSM, plants were grown in purpose-built chambers, constructed using a microscope slide and long cover glass with a 4 mm spacer between them on 3 sides and an opening at the top. The spacer was glued to the slide and cover glass using Araldite glass and ceramic adhesive (Huntsman International). The chambers were covered with aluminium foil on the outside during growth to exclude light from the roots. Foil was removed immediately before imaging. Before imaging, transparent soil was saturated with MSR containing 13% (w/v) sorbitol or 98% Percoll (Sigma). The refractive index of the solution matches the refractive index of the Nafion particles used here to provide complete transparency in the substrate. Sulphorhodamine B (Sigma) at 1 µg ml^−1^ was used to dye the particles in situ before imaging. A Leica TCS SP2 confocal laser scanning microscope and objective lenses 2.5×/0.07, 10×/0.30, 20×/0.50, 40×/0.80 and 63×/0.90 were used to obtain the confocal scans.

### Data Analysis

Analysis of variance and multiple comparisons were carried out using Genstat 13^th^ Edition (VSN International Ltd.). Sigmaplot 12 (Syststat Software, Inc.) was used for non-linear regression. Avizo software (VSG) was used for visualisation of CLSM images. Image analysis was carried out using Mevislab [43] and Fiji Software [44]. Root tracking used an algorithm by Friman et al [45]. Skeletonization and edge detection was carried out using the standard Mevislab algorithms developed respectively by Milo Hindennach and Olaf Konrad and Wolf Spindler.

## Supporting Information

Figure S1 **Snapshots of volume renderings of confocal scans of** ***Arabidopsis thaliana*** **roots expressing GFP in plasma membranes (grey) in transparent soil with sulphorhdamine-B-dyed particles (orange).** A. Lateral root emerging from primary root. Scale bar represents 170 µm. B. Section of primary root and root hairs in contact with Nafion particle. Scale bar represents 40 µm.
(TIF)

Video S1 **In situ 3D image of branched** ***Arabidopsis thaliana*** **roots expressing GFP in plasma membranes (green) in transparent soil with sulphorhdamine-B-dyed particles (red).**
(MPG)

Video S2 **In situ 3D image of** ***Arabidopsis thaliana*** **root with emrging lateral root expressing GFP in plasma membranes (green) in transparent soil with sulphorhdamine-B-dyed particles (orange).**
(MPG)

Video S3 **In situ 3D image of** ***Arabidopsis thaliana*** **root with root hairs expressing GFP in plasma membranes (green) with Nafion particle of transparent soil (orange).**
(MPG)

Video S4 (MPG)
