# Supplementary figures and images for: Transparent Soil for Imaging the Rhizosphere
Source: PLoS One. 2012 Sep 11;7(9):e44276. doi: 10.1371/journal.pone.0044276 (PMC3439476; doi:10.1371/journal.pone.0044276)

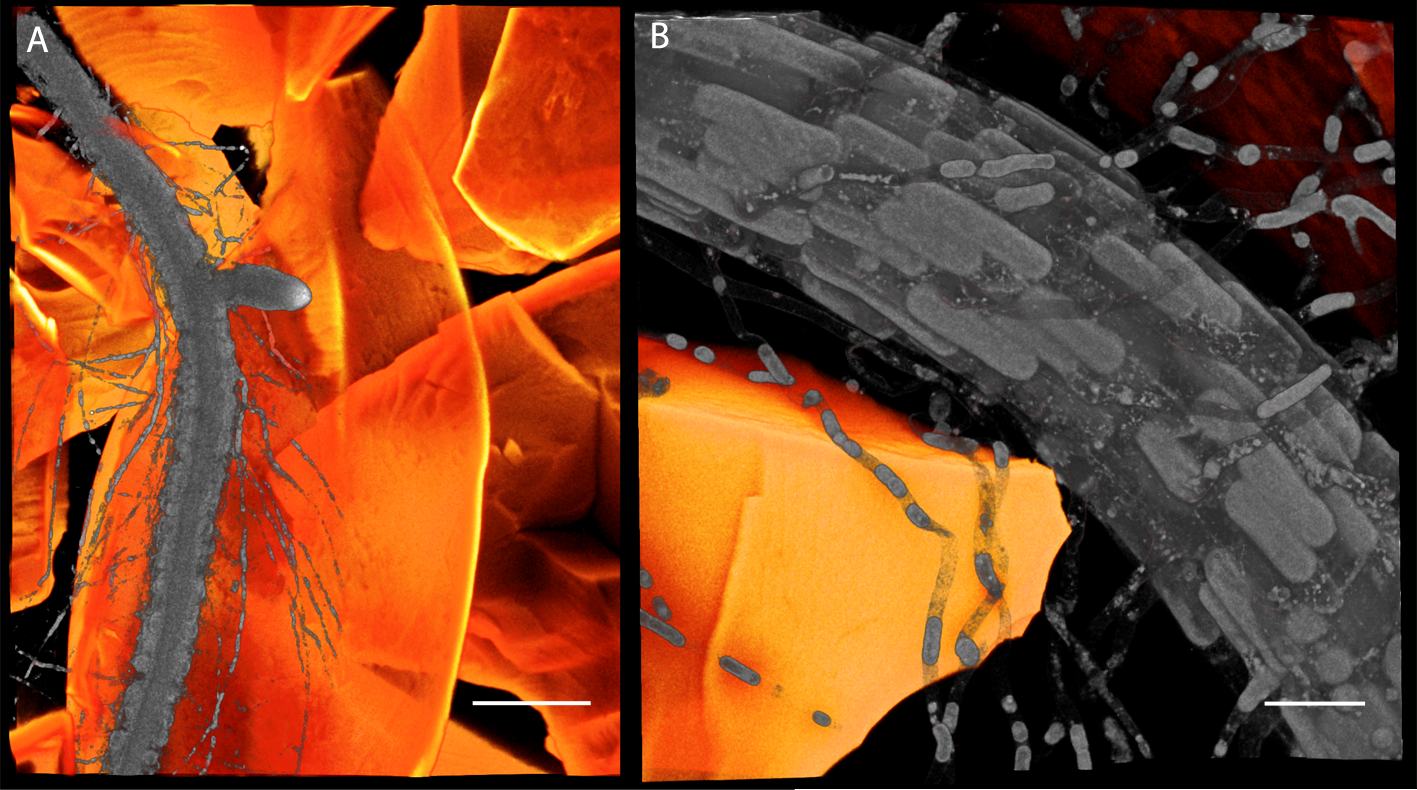

Supplement: Figure S1 — Snapshots of volume renderings of confocal scans of Arabidopsis thaliana roots expressing GFP in plasma membranes (grey) in transparent soil with sulphorhdamine-B-dyed particles (orange). A. Lateral root emerging from primary root. Scale bar represents 170 µm. B. Section of primary root and root hairs in contact with Nafion particle. Scale bar represents 40 µm. (TIF) [file pone.0044276.s001.tif]
